# rstoolbox - a Python library for large-scale analysis of computational protein design data and structural bioinformatics

**DOI:** 10.1186/s12859-019-2796-3

**Published:** 2019-05-15

**Authors:** Jaume Bonet, Zander Harteveld, Fabian Sesterhenn, Andreas Scheck, Bruno E. Correia

**Affiliations:** 1 Institute of Bioengineering, École Polytechnique Fédérale de Lausanne, CH-1015 Lausanne, Switzerland; 2 Swiss Institute of Bioinformatics (SIB), CH-1015 Lausanne, Switzerland

**Keywords:** rstoolbox, Computational protein design, Protein structural metrics, Scoring, Data analysis

## Abstract

**Background:**

Large-scale datasets of protein structures and sequences are becoming ubiquitous in many domains of biological research. Experimental approaches and computational modelling methods are generating biological data at an unprecedented rate. The detailed analysis of structure-sequence relationships is critical to unveil governing principles of protein folding, stability and function. Computational protein design (CPD) has emerged as an important structure-based approach to engineer proteins for novel functions. Generally, CPD workflows rely on the generation of large numbers of structural models to search for the optimal structure-sequence configurations. As such, an important step of the CPD process is the selection of a small subset of sequences to be experimentally characterized. Given the limitations of current CPD scoring functions, multi-step design protocols and elaborated analysis of the decoy populations have become essential for the selection of sequences for experimental characterization and the success of CPD strategies.

**Results:**

Here, we present the rstoolbox, a Python library for the analysis of large-scale structural data tailored for CPD applications. rstoolbox is oriented towards both CPD software *users* and *developers*, being easily integrated in analysis workflows. For *users*, it offers the ability to profile and select decoy sets, which may guide multi-step design protocols or for follow-up experimental characterization. rstoolbox provides intuitive solutions for the visualization of large sequence/structure datasets (e.g. logo plots and heatmaps) and facilitates the analysis of experimental data obtained through traditional biochemical techniques (e.g. circular dichroism and surface plasmon resonance) and high-throughput sequencing. For CPD software *developers*, it provides a framework to easily benchmark and compare different CPD approaches. Here, we showcase the rstoolbox in both types of applications.

**Conclusions:**

rstoolbox is a library for the evaluation of protein structures datasets tailored for CPD data. It provides interactive access through seamless integration with IPython, while still being suitable for high-performance computing. In addition to its functionalities for data analysis and graphical representation, the inclusion of rstoolbox in protein design pipelines will allow to easily standardize the selection of design candidates, as well as, to improve the overall reproducibility and robustness of CPD selection processes.

## Background

The fast-increasing amounts of biomolecular structural data are enabling an unprecedented level of analysis to unveil the principles that govern structure-function relationships in biological macromolecules. This wealth of structural data has catalysed the development of computational protein design (CPD) methods, which has become a popular tool for the structure-based design of proteins with novel functions and optimized properties [1]. Due to the extremely large size of the sequence-structure space [2], CPD is an NP-hard problem [3]. Two different approaches have been tried to address this problem: deterministic and heuristic algorithms.

Deterministic algorithms are aimed towards the search of a single-best solution. The OSPREY design suite, which combines Dead-End Elimination theorems combined with A* search (DEE/A*) [4], is one of the most used software relying on this approach. By definition, deterministic algorithms provide a sorted, continuous list of results. This means that, according to their energy function, one will find the best possible solution for a design problem. Nevertheless, as energy functions are not perfect, the selection of multiple decoys for experimental validation is necessary [5, 6]. Despite notable successes [7–9], the time requirements for deterministic design algorithms when working with large proteins or de novo design approaches limits their applicability, prompting the need for alternative approaches for CPD.

Heuristic algorithms, such as those based on Monte Carlo (MC) sampling [10], use stochastic sampling methods together with scoring functions to guide the structure and sequence exploration towards an optimized score. These algorithms have the advantage of sampling the sequence-structure space within more reasonable time spans, however, they do not guarantee that the final solutions reached the global minimum [11]. Heuristic CPD workflows address this shortcoming in two ways: I) extensive sampling generating large decoy sets; II) sophisticated ranking and filtering schemes to discriminate and identify the best solutions. This general approach is used by the Rosetta modelling suite [12], one of the most widespread CPD tools.

For Rosetta, as with other similar approaches, the amount of sampling necessary scales with the degrees of freedom (conformational and sequence) of a particular CPD task. Structure prediction simulations such as *ab initio* or docking may require to generate up to 10^6^ decoys to find acceptable solutions [13, 14]. Similarly, for different design problems the sampling scale has been estimated. Sequence design using static protein backbones (fixed backbone design) [15] may reach sufficient sampling within hundreds of decoys. Protocols that allow even limited backbone flexibility, dramatically increase the search space, requiring 10^4^ to 10^6^ decoys, depending on the number of residues for which sequence design will be performed. Due to the large decoy sets generated in the search for the best design solution, as well as the specificities of each design case, researchers tend to either generate one-time-use scripts or analysis scripts provided by third parties [16]. In the first case, these solutions are not standardized and its logic can be difficult to follow. In the second case, these scripts can be updated over time without proper back-compatibility control. As such, generalized tools to facilitate the management and analysis of the generated data are essential to CPD pipelines.

Here, we present rstoolbox, a Python library to manage and analyse designed decoy sets. The library presents a variety of functions to produce multi-parameter scoring schemes and compare the performance of different CPD protocols. The library can be accessed by users within three levels of expertise: a collection of executables for designers with limited coding experience, interactive interfaces such as Ipython [17] for designers with basic experience in data analysis (i.e. pandas [18]), and a full-fledge API to be used by developers to benchmark and optimize new CPD protocols. This library was developed for direct processing of Rosetta output files, but its general architecture makes it easily adaptable to other CPD software. The applicability of the tools developed expands beyond the analysis of CPD data making it suitable for general structural bioinformatics problems (see extended_example notebook in the code’s repository). Thus, we foresee that rstoolbox may provide a number of useful functionalities for the broad structural bioinformatics community.

## Implementation

**Fig. 1:**
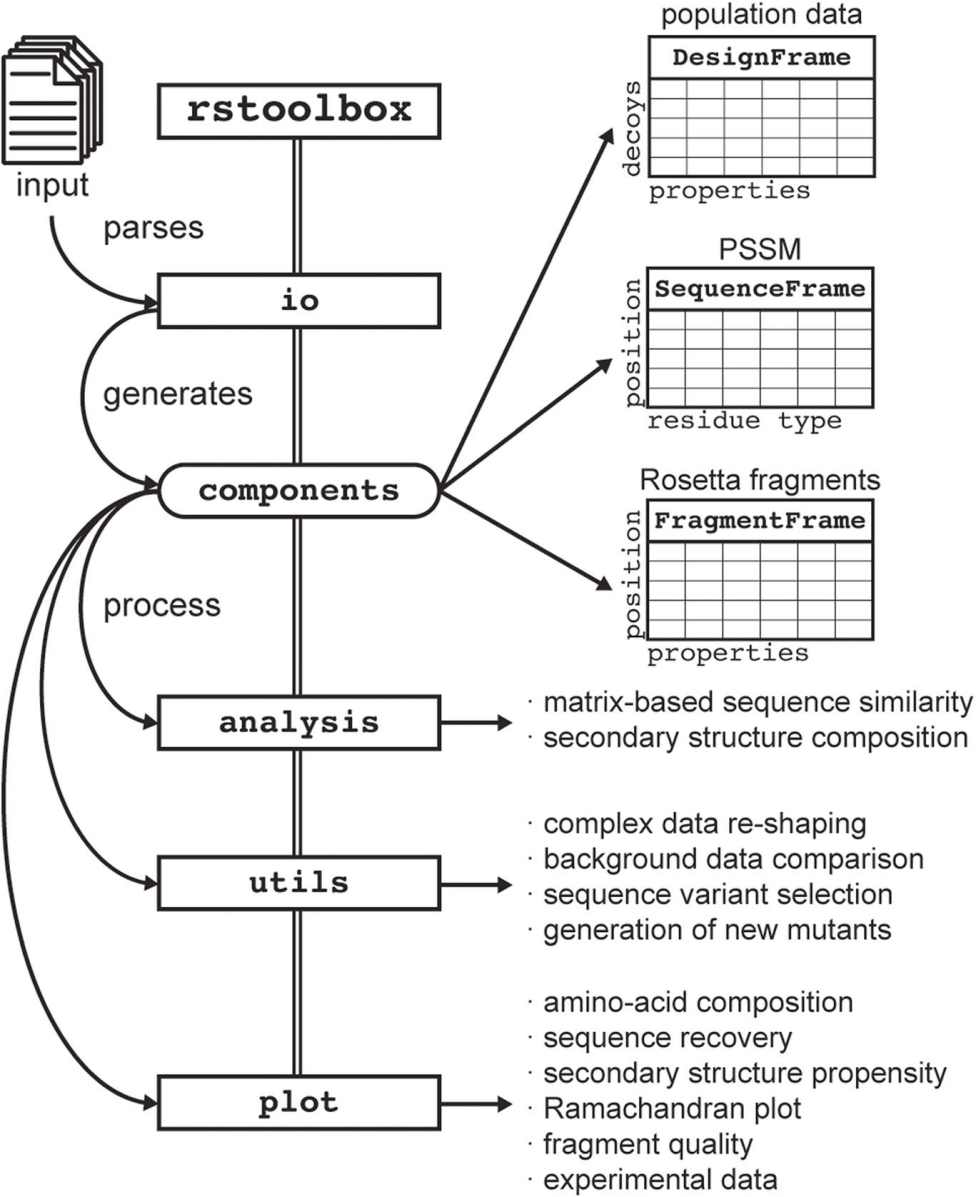
rstoolbox library architecture. The io module contains functions for parsing the input data. The input functions in io generate one of the three data containers defined in the components module: DesignFrame for decoy populations, SequenceFrame for per-position amino acid frequencies and FragmentFrame for Rosetta’s fragments. The other three modules analysis, utils and plot, provide all the functions to manipulate, process and visualize the data stored in the different components

rstoolbox has been implemented extending from pandas [18], one of the most established Python libraries for high-performance data analysis. The rstoolbox library architecture is composed of 4 functional modules (Fig. 1): I) rstoolbox.io - provides read/write functions for multiple data types, including computational design simulations and experimental data, in a variety of formats; II) rstoolbox.analysis - provides functions for sequence and structural analysis of designed decoys; III) rstoolbox.plot – plotting functionalities that include multiple graphical representations for protein sequence and structure features, such as logo plots [19], Ramachandran distributions [20], sequence heatmaps and other general plotting functions useful for the analysis of CPD data; IV) rstoolbox.utils – helper functions for data manipulation and conversion, comparison of designs with native proteins and the creation of amino acid profiles to inform further iterations of the design process.

**Table 1:** Sample code for the evaluation of protein backbone dihedral angles and fragment quality

Action	Code Sample
Load	**import rstoolbox as rs** import matplotlib.pyplot as plt import seaborn as sns
Read	*# With Rosetta installed, a single structure is scored. The* *# function will return multiple score terms, sequence,* *# secondary structure and phi/psi angles.* ref = **rs.io.get_sequence_and_structure**(‘1kx8_d2.pdb’)
	*# Loading Rosetta fragments* seqfrags = **rs.io.parse_rosetta_fragments**(‘seq.200.9mers’) *# With Rosetta, structural similarity of the fragments can be measured* seqfrags = seqfrags. **add_quality_measure** (None, ‘mota_1kx8_d2.pdb’) strfrags = **rs.io.parse_rosetta_fragments**(‘str.200.9mers’) strfrags = strfrags. **add_quality_measure** (None, ‘mota_1kx8_d2.pdb’)
	*# Loading* *ab initio* *data* abseq = **rs.io.parse_rosetta_file**(‘abinitio_seqfrags.minsilent.gz’) abstr = **rs.io.parse_rosetta_file**(‘abinitio_strfrags.minsilent.gz’)
Plot	fig = plt.figure(figsize = (170 / 25.4, 170 / 25.4)) grid = (3, 6)
	*# There are 4 flavours of Ramachandran plots available depending on the* *# targeted residues: GENERAL, GLY, PRE-PRO and PRO.* ax1 = plt.subplot2grid(grid, (0, 0), colspan = 2) *# Ramachandran is plotted for a single decoy (selected as parameter 1).* *# As a decoy can contain multiple chains, the chain identifier is an* *# ubiquitous attribute in multiple functions of the library.* **rs.plot.plot_ramachandran_single** (ref.iloc[0], ‘A’, ax1) ax1 = plt.subplot2grid(grid, (0, 2), fig = fig, colspan = 2) **rs.** **plot.plot_ramachandran_single** (ref.iloc[0], ‘A’, ax1, ‘PRE-PRO’) ax1 = plt.subplot2grid(grid, (0, 4), colspan = 2) **rs.plot.plot_ramachandran_single** (ref.iloc[0], ‘A’, ax1, ‘PRO’)
	*# Show RMSD match of fragments to the corresponding sequence for a* *# selected region* ax1 = plt.subplot2grid(grid, (1, 0), colspan = 3) ax2 = plt.subplot2grid(grid, (1, 3), colspan = 3, sharey = ax1) **rs.plot.plot_fragments** (seqfrags. **slice_region** (21, 56), strfrags.**slice_region**(21, 56), ax1, ax2) **rs.utils.add_top_title** (ax1, ‘sequence-based 9mers’) **rs.utils.add_top_title** (ax2, ‘structure-based 9mers’)
	*# DataFrames can directly work with widely spread plotting functions* ax1 = plt.subplot2grid(grid, (2, 0), colspan = 3) sns.scatterplot(x = “rms”, y = “score”, data = abseq, ax = ax1) ax2 = plt.subplot2grid(grid, (2, 3), colspan = 3, sharey = ax1, sharex = ax1) sns.scatterplot(x = “rms”, y = “score”, data = abstr, ax = ax2) **rs.utils.add_top_title** (ax1, ‘sequence-based fragments’) **rs.utils.add_top_title** (ax2, ‘structure-based fragments’)
	plt.tight_layout() plt.savefig(‘BMC_Fig2.png’, dpi = 300)

The code shows how to combine structural data obtained from a protein structure file with fragment quality evaluated by Rosetta and *ab initio* simulations. Code comments are presented in italics while functions from the rstoolbox are highlighted in bold. Styling commands are skipped to facilitate reading, but can be found in the repository’s notebook.

Additionally, rstoolbox contains 3 table-like data containers defined in the rstoolbox.components module (Fig. 1): I) DesignFrame - each row is a designed decoy and the columns represent decoy properties, such as, structural and energetic scores, sequence, secondary structure, residues of interest among others; II) SequenceFrame - similar to a position-specific scoring matrix (PSSM), obtained from the DesignFrame can be used for sequence and secondary structure enrichment analysis; III) FragmentFrame - stores fragment sets, a key element in Rosetta’s *ab initio* folding and loop closure protocols. Derived from pandas.DataFrame [18], all these objects can be casted from and to standard data frames, making them compatible with libraries built for data frame analysis and visualization.

The DesignFrame is the most general data structure of the library. It allows fast sorting and selection of decoys through different scores and evaluation of sequence and structural features. It can be filled with any tabulated, csv or table-like data file. Any table-formatted data can be readily input, as the generation of parsers and integration into the rstoolbox framework is effortless, providing easy compatibility with other CPD software packages, in addition to Rosetta. Currently, rstoolbox provides parsers for FASTA files, CLUSTALW [21] and HMMER [22] outputs, Rosetta’s json and silent files (Fig. 1).

The components of the library can directly interact with most of the commonly used Python plotting libraries such as matplotlib [23] or seaborn [24]. Additional plotting functions, such as logo and Ramachandran plots, are also present to facilitate specific analysis of CPD data. As mentioned, this library has been developed primarily to handle Rosetta outputs and thus, rstoolbox accesses Rosetta functions to extract structural features from designed decoys (e. g. backbone dihedral angles). Nevertheless, many of the rstoolbox’s functionalities are independent of a local installation of Rosetta. rstoolbox is configured with a continuous integration system to guarantee a robust performance upon the addition of new input formats and functionalities. Testing covers more than 80% of the library’s code, excluding functions that have external dependencies from programs like Rosetta [12], HMMER [22] or CLUSTALW [21]. To simplify its general usage, the library has a full API documentation with examples of common applications and can be directly installed with PyPI (pip install rstoolbox).

## Results

### Analysis of protein backbone features

**Fig. 2:**
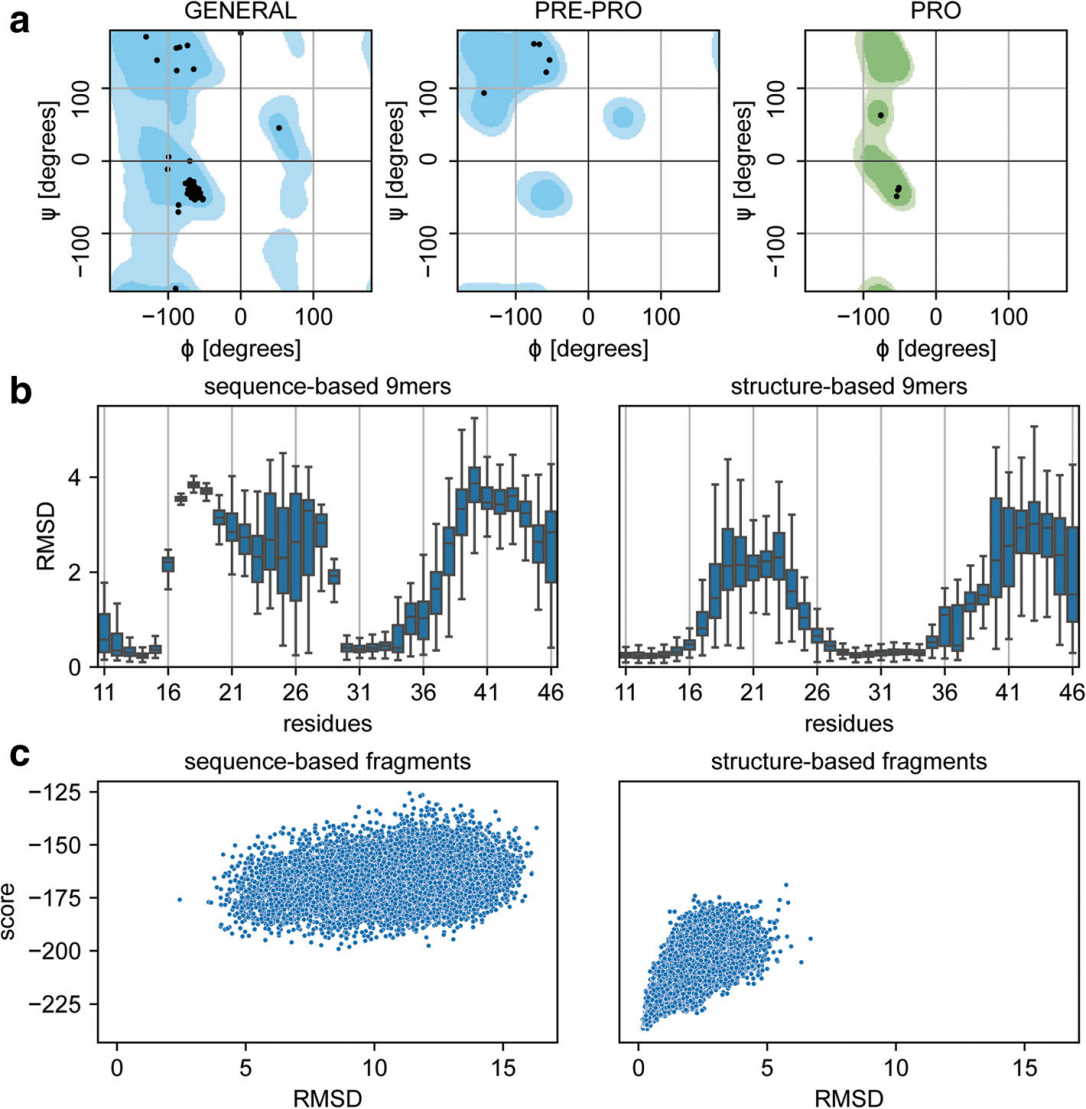
Ramachandran plots and fragment quality profiles. Assessment of fragments generated using distinct input data and their effect on Rosetta *ab initio* simulations. With the exception of the panel identifiers, the image was created with the code presented in Table 1. **a** Ramachandran distribution of a query structure. **b** Fragment quality comparison between sequence- and structure-based fragments. The plot shows a particular region of the protein for which sequence-based fragments present much larger structural deviations than structure-based fragments in comparison with the query protein. **c** Rosetta *ab initio* simulations performed with sequence- (left) or structure-based (right) fragments. Fragments with a better structural mimicry relative to the query structure present an improved folding funnel

A typical metric to assess the quality of protein backbone conformations is by comparison of the backbone dihedral angles with those of the Ramachandran distributions [20]. Such evaluation is more relevant in CPD strategies that utilize flexible backbone sampling, which have become increasingly used in the field (e.g. loop modelling [25], de novo design [26]). A culprit often observed in designs generated using flexible backbone sampling is that the modelled backbones present dihedral angles in disallowed regions of the Ramachandran distributions, meaning that such conformations are likely to be unrealistic. To identify these problematic structures, rstoolbox provides functions to analyse the dihedral angles of decoy sets and represent them in Ramachandran plots (Table 1, Fig. 2a).

Furthermore, structural prediction has also become an integral part of many CPD workflows [27]. Here, one evaluates if the designed sequences have energetic propensity to adopt the desired structural conformations. A typical example where prediction is recurrently used as a criterion to select the best designed sequences is on de novo design. To assess the ability of novel sequences to refold to the target structures, the Rosetta *ab initio* protocol is typically used [13]. Importantly, the quality of the predictions is critically dependent on the fragment sets provided as input as they are used as local building blocks to assemble the folded three-dimensional structures. The local structural similarity of the fragments to the target structure largely determines the quality of the sampling of the *ab initio* predictions. rstoolbox provides analysis and plotting tools to evaluate the similarity of fragment sets to a target structure (Fig. 2b). In Fig. 2c the impact of distinct fragment sets in *ab initio* predictions is shown where a clear folding funnel is visible for fragments with high structural similarity. This tool can also be useful for structural prediction applications to profile the quality of different fragment sets.

### Guiding iterative CPD workflows

Many CPD workflows rely on iterative approaches in which multiple rounds of design are performed and each generation of designs is used to guide the next one.

**Fig. 3:**
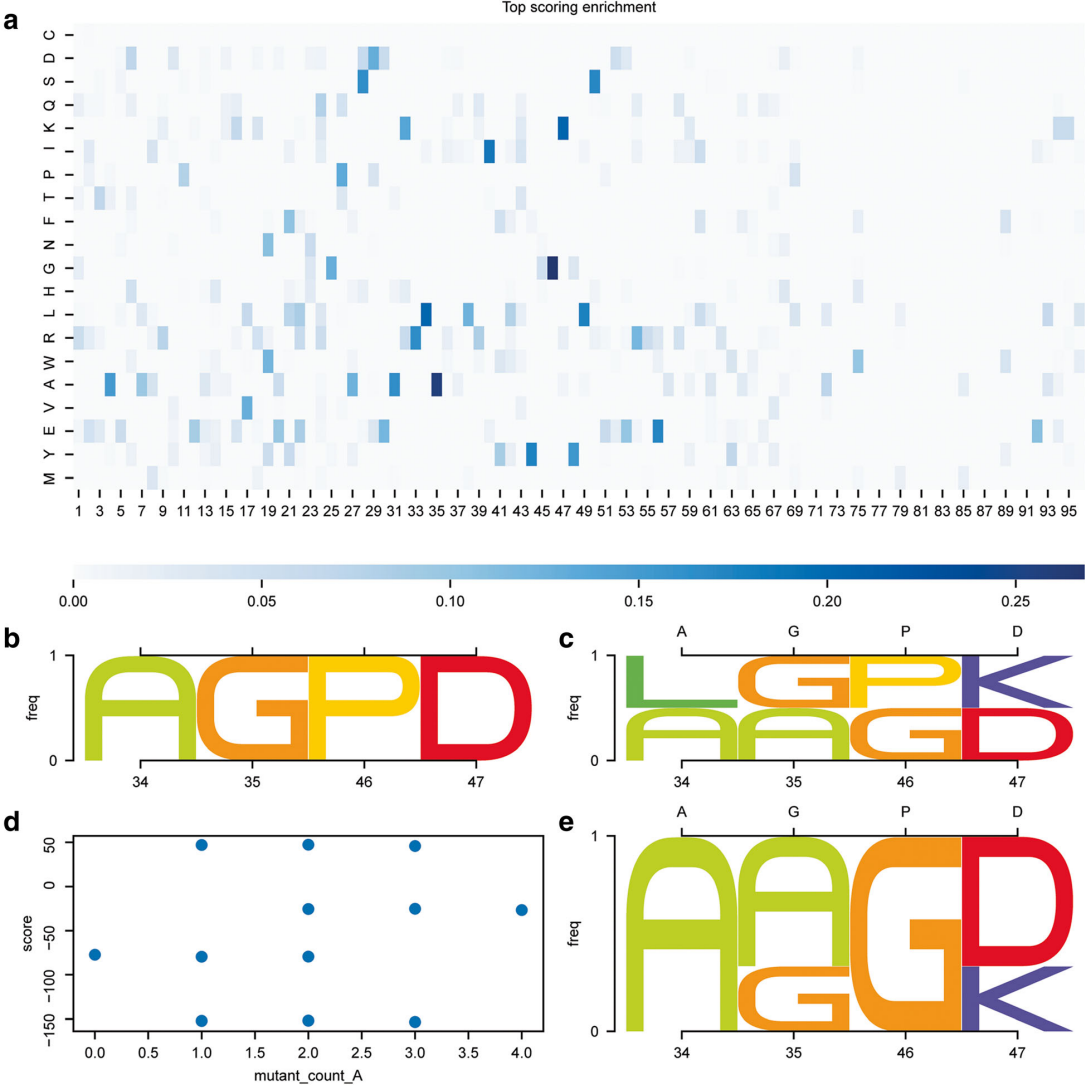
Guiding iterative design pipelines. Information retrieved from decoy populations can be used to guide following generations of designs. With the exception of the panel identifiers, the image was directly created with the code presented in Table 2. **a** Mutant enrichment from comparison of the design on top 5% by score and the overall population. Positions 34, 35, 46 and 47 present a 20% enrichment of certain residue types over the whole population and are selected as positions of interest. **b** Residue types for the positions of interest in the decoy selected as template of the second generation. **c** Upon guided mutagenesis, we obtain a total of 16 decoys including the second-generation template. We can observe that the overrepresented residues shown in A are now present in the designed population. Upper x axis shows the original residue types of the template. **d** Combinatorial targeted mutagenesis yields 16 new designs, three of which showed an improved total score relative to the second-generation template (mutant_count_A is 0). **e** The three best scoring variants show mutations such as P46G which seem to be clearly favorable for the overall score of the designs. Upper x axis shows the original residue types of the template

The rstoolbox presents a diversity of functions that aid this process and perform tasks from selecting decoys with specific mutations of interest, to those that define residue sets for instance based in position weight matrices (generate_mutants_from_matrix()). When redesigning naturally occurring proteins, it also presents a function to generate reversions to wild-type residues (generate_wt_reversions()) to generate the best possible design with the minimal number of mutations. These functions will directly execute Rosetta, if installed in the system, but can also be used to create input files to run the simulations in different software suits. Code example for these functionalities is shown in Table 2. The result of the code is depicted on Fig. 3.

**Table 2:** Sample code to guide iterative CPD workflows

Action	Code Sample
Load	**import rstoolbox as rs** import matplotlib.pyplot as plt import seaborn as sns
Read	*# Load design population. A description dictionary can be provided to alter the* *# information loaded from the silent file. In this case, we load all the* *# sequence information available for all possible chains in the decoys.* df = **rs.io.parse_rosetta_file**(‘1kx8gen2.silent.gz’, {‘sequence’: ‘*’})
	*# Select the top 5% designs by score and obtain the residues* *# overrepresented by more than 20%* df_top = df[df[‘score’] < df[‘score’].quantile(0.05)] freq_top = **rs.analysis.sequential_frequencies**(df_top, ‘A’, ‘sequence’, ‘protein’) freq_all = df **.sequence_frequencies** (‘A’) *# shortcut to utils.sequential_frequencies* freq_diff = (top - freq) muts = freq_diff[(freq_diff.T > 0.20).any()].idxmax(axis = 1) muts = list(zip(muts.index, muts.values))
	*# Select the best scored sequence that does NOT contain ANY of those residues* pick = df **.get_sequence_with** (‘A’, muts, confidence = 0.25, invert = True).sort_values(‘score’).iloc[:1] *# Setting a reference sequence in a DesignFrame allows to use this sequence as* *# source for mutant generation and sequence comparison, amongst others.* seq = pick.iloc[0 **].get_sequence** (‘A’) pick **.add_reference_sequence** (‘A’, seq)
	*# Generate mutants based on the identified overrepresented variants:* *# 1. Create a list with positions and residue type expected in each position* muts = [(muts[i][0], muts[i] [1] + seq[muts[i][0] - 1]) for i in range (len(muts))] *# 2* Generate a DesignFrame containing the new expected sequences variants = pick **.generate_mutant_variants** (‘A’, muts) variants **.add_reference_sequence** (‘A’, seq) *# 3*. Generate the resfiles that will guide the mutagenesis variants = variants **.make_resfile** (‘A’, ‘NATAA’, ‘mutants.resfile’) *# 4. With Rosetta installed, we can automatically run those resfiles.* variants = variants **.apply_resfile** (‘A’, ‘variants.silent’) variants = variants **.identify_mutants** (‘A’)
Plot	fig = plt.figure(figsize = (170 / 25.4, 170 / 25.4)) grid = (3, 4)
	*# Visualize overrepresented residues in the top 5%* ax = plt.subplot2grid(grid, (0, 0), colspan = 4, rowspan = 4) cbar_ax = plt.subplot2grid(grid, (4, 0), colspan = 4, rowspan = 1) sns.heatmap(freq_diff.T, ax = ax, vmin = 0, cbar_ax = cbar_ax) **rs.utils.add_top_title** (ax, ‘Top scoring enrichment’)
	*# Compare query positions: initial sequence* vs. *mutant generation* ax = plt.subplot2grid(grid, (5, 0), colspan = 2, rowspan = 2) key_res = [mutants[0] for mutants in muts] **rs.plot.logo_plot_in_axis** (pick, ‘A’, ax = ax, _residueskr) ax = plt.subplot2grid(grid, (5, 2), colspan = 2, rowspan = 2) **rs.plot.logo_plot_in_axis** (variants, ‘A’, ax = ax, key_residues = kr)
	*# Check which mutations perform better* ax = plt.subplot2grid(grid, (7, 0), colspan = 2, rowspan = 3) sns.scatterplot(‘mutant_count_A’, ‘score’, data = variants, ax = ax) *# Show distribution of best performing decoys* ax = plt.subplot2grid(grid, (7, 2), fig = fig, colspan = 2, rowspan = 3) **rs.plot.logo_plot_in_axis** (variants.sort_values(‘score’).head(3), ‘A’, ax = ax, key_residues = kr) plt.tight_layout() plt.savefig(‘BMC_Fig3.png’, dpi = 300)

This example shows how to find overrepresented residue types for specific positions in the top 5% scored decoys of a design population, and use those residue types to bias the next design generation, thus creating a new, enriched second generation population. Code comments are presented in italics while functions from rstoolbox are highlighted in bold. Styling commands are skipped to facilitate reading, but can be found in the repository’s notebook.

rstoolbox allows the user to exploit the data obtained from the analysis of designed populations in order to bias following design rounds. When using rstoolbox, this process is technically simple and clear to other users, which will improve the comprehension and reproducibility of iterative design pipelines.

### Evaluation of designed proteins

**Fig. 4:**
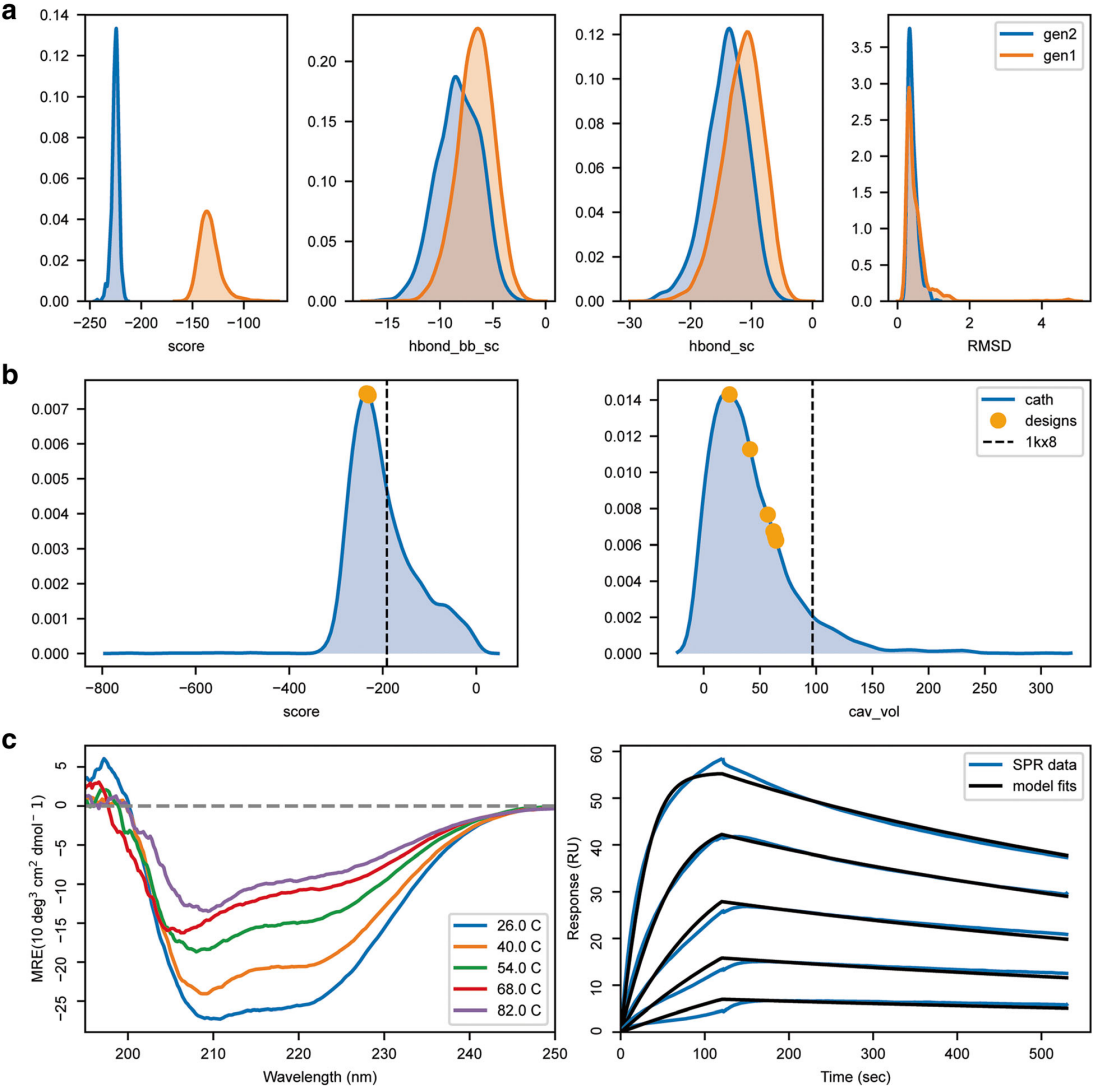
Multi-stage design, comparison with native proteins and representation of experimental data for 1kx8-based epitope-scaffold. Analysis of the two-step design pipeline, followed by a comparison of the distributions obtained for native proteins and the designs and plotting of biochemical experimental data. With the exception of the panel identifiers, the image was directly created with the code presented in Table 3. **a** Comparison between the first (orange) and the second (blue) generation of designs. score – shows the Rosetta energy score; hbond_bb_sc – quantifies the hydrogen bonds between backbone and side chain atoms; hbond_sc - quantifies the hydrogen bonds occurring between side chain atoms; RMSD – root mean square deviation relative to the original template. Second-generation designs showed minor improvements on backbone hydrogen bonding and a substantial improvement in overall Rosetta Energy. **b** Score and cavity volume for the selected decoys in comparison with structures of CATH [31] domains of similar size. The vertical dashed black line represents the score and cavity volume of the original 1kx8 after minimization, highlighting the improvements relative to the original scaffold. **c** Circular Dichroism and Surface Plasmon Resonance data for the best design shows a well folded helical protein that binds with high affinity to the expected target

Recently, we developed the Rosetta FunFolDes protocol, which was devised to couple conformational folding and sequence design [28]. FunFolDes was developed to insert functional sites into protein scaffolds and allow for full-backbone flexibility to enhance sequence sampling. As a demonstration of its performance, we designed a new protein to serve as an epitope-scaffold for the Respiratory Syncytial Virus site II (PDB ID: 3IXT [29]), using as scaffold the A6 protein of the Antennal Chemosensory system from *Mamestra brassicae* (PDB ID: 1KX8 [30]). The designs were obtained in a two-stage protocol, with the second generation being based on the optimization of a small subset of first-generation decoys. The code presented in Table 3 shows how to process and compare the data of both generations. Extra plotting functions to represent experimental data obtained from the biochemical characterization of the designed proteins is also shown. The result of this code is represented in Fig. 4.

**Table 3:** Sample code for the evaluation of a multistep design pipeline

Action	Code Sample
Load	**import rstoolbox as rs** import matplotlib.pyplot as plt
Read	*# With Rosetta installed, scoring can be run for a single structure* baseline = **rs.io.get_sequence_and_structure**(‘1kx8.pdb’, minimize = True) slen = len(baseline.iloc[0 **].get_sequence** (‘A’)) *# Pre-calculated sets can also be loaded to contextualize the data* *# 70% homology filter* cath = **rs.utils.load_refdata**(‘cath’, 70) *# Length in a window of 10 residues around expected design length* cath = cath[(cath[‘length’] > = slen - 5) & (cath[‘length’] < = slen + 5)] *# Designs were performed in two rounds* gen1 = **rs.io.parse_rosetta_file**(‘1kx8_gen1.designs’) gen2 = **rs.io.parse_rosetta_file**(‘1kx8_gen2.designs’) *# Identifiers of selected decoys:* decoys = [‘d1’, ‘d2’, ‘d3’, ‘d4’, ‘d5’, ‘d6’] *# Load experimental data for d2 (best performing decoy)* df_cd = **rs.io.read_CD**(‘1kx8_d2/CD’, model = ‘J-815’) df_spr = **rs.io.read_SPR**(‘1kx8_d2/SPR.data’)
Plot	fig = plt.figure(figsize = (170 / 25.4, 170 / 25.4)) grid = (3, 4) *# Compare scores between the two generations* axs = **rs.plot.multiple_distributions**(gen2, fig, (3, 4), values = [‘score’, ‘hbond_bb_sc’, ‘hbond_sc’, ‘rmsd’], refdata = gen1, violins = False, showfliers = False)
	# See how the selected decoys fit into domains of similar size qr = gen2[gen1[‘description’].isin(decoys)] axs = **rs.plot.plot_in_context**(qr, fig, (3, 2), cath, (1, 0), [‘score’, ‘cav_vol’]) axs[0].axvline(baseline.iloc[0][‘score’], color = ‘k’, linestyle = ‘--’) axs[1].axvline(baseline.iloc[0][‘cavity’], color = ‘k’, linestyle = ‘--’)
	*# Plot experimental validation data* *ax = plt.subplot2grid(grid, (2, 0), fig = fig, colspan = 2)* **rs.plot.plot_CD** (df_cd, ax, sample = 7) *ax = plt.subplot2grid(grid, (2, 2), fig = fig, colspan = 2)* **rs.plot.plot_SPR** (df_spr, ax, fitcolor = ‘black’)
	plt.tight_layout() plt.savefig(‘BMC_Fig4.png’, dpi = 300)

The code shows how to combine the data from multiple Rosetta simulations and assess the different features between two design populations in terms of scoring as well as the comparison between the final designs and the initial structure template. Code comments are presented in italics while functions from the rstoolbox are highlighted in bold. Styling commands are skipped to facilitate reading, but can be found in the repository’s notebook.

### Benchmarking design protocols

**Fig. 5:**
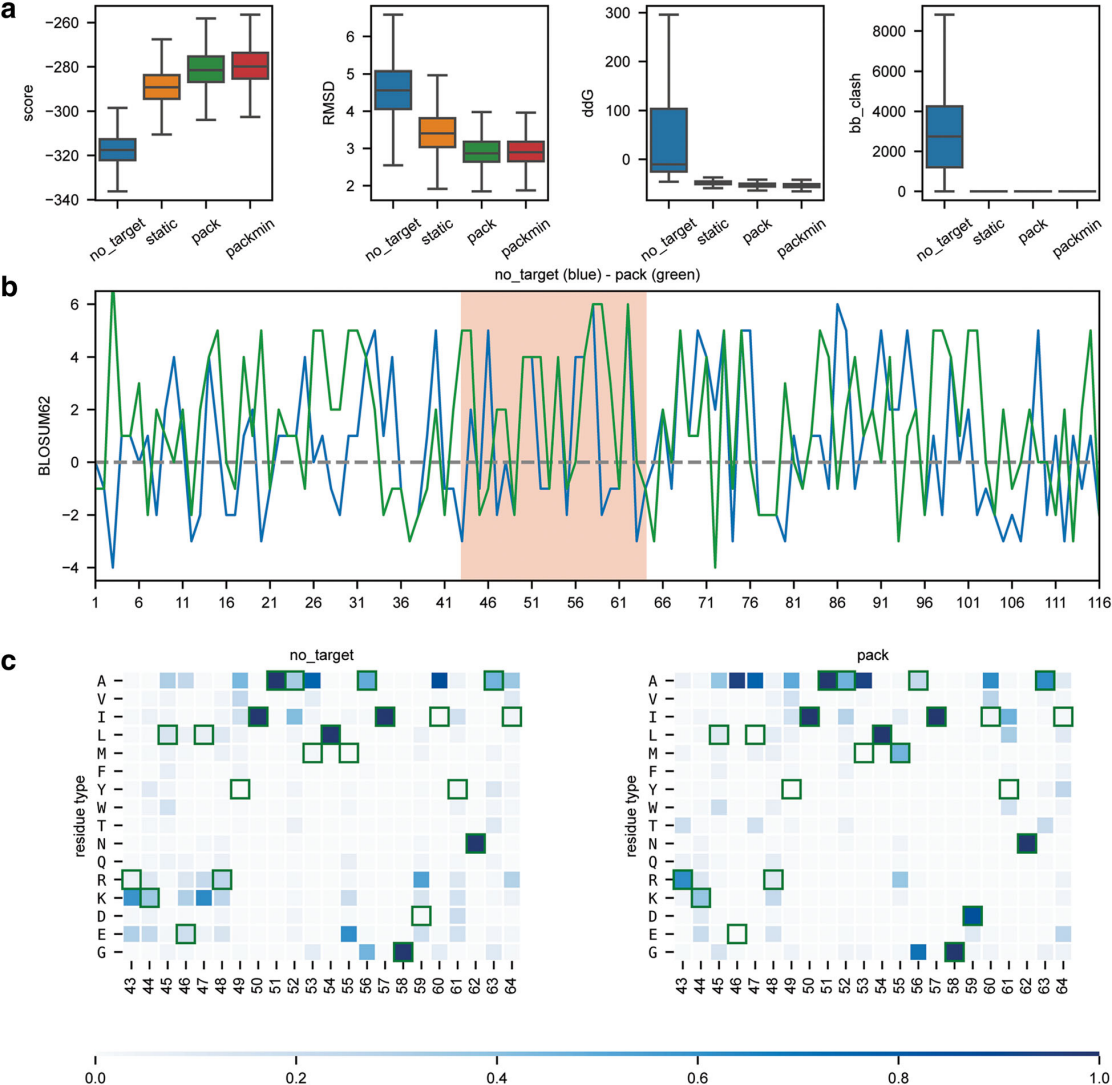
Comparison and benchmarking of different design protocols. Representation of the results obtained using four different design protocols. With the exception of the panel identifiers, the image was directly created with the code presented in Table 4. **a** Representation of four scoring metrics in the design of a new protein binder. score – shows the overall Rosetta score; RMSD – root mean square deviation relative to BINDI; ddG –Rosetta energy for the interaction between two proteins; bb_clash - quantifies the backbone clashes between the binder and the target protein; **b** BLOSUM62 positional sequence score for the top design of the *no_target* (blue) and *pack* (green) design populations showcases how to analyse and compare individual decoys. The higher the value, the more likely two residue types (design vs. BINDI) are to interchange within evolutionary related proteins. Special regions of interest can be easily highlighted, as for instance the binding region (highlighted in salmon). **c** Population-wide analysis of the sequence recovery of the binding motif region for *no_target* and *pack* simulations*.* Darker shades of blue indicate a higher frequency and green frames indicate the reference residue type (BINDI sequence). This representation shows that the *pack* population explores more frequently residue types found in the BINDI design in the region of the binding motif

One of the main novelties of FunFolDes was the ability to include a binding partner during the folding-design simulations. This feature allows to bias the design simulations towards productive configurations capable of properly displaying the functional motif transplanted to the scaffold. To assess this new feature, we used as a benchmark test the previously computationally designed protein BINDI, a 3-helix bundle that binds to BHRF1 [32]. We performed simulations under four different conditions: *no-target* (binding-target absent), *static* (binding-target without conformational freedom), *pack* (binding-target with side-chain repacking) and *packmin* (binding-target with side chain repacking and backbone minimization) and evaluated the performance of each simulation. Specifically, we analysed how the design populations performed regarding energetic sampling (Fig. 5a) and the mimicry of BINDI’s conformational shift from the original scaffold (Fig. 5a). In addition, we quantified the sequence recovery relative to the experimentally characterized BINDI sequence (Fig. 5b and c). Table 4 exemplifies how to easily load and combine the generated data and create a publication-ready comparative profile between the four different approaches (Fig. 5).

**Table 4:** Sample code for the comparison between 4 different decoy populations

Action	Code Sample
Load	import pandas as pd **import rstoolbox as rs** import matplotlib.pyplot as plt
Read	df = [] *# With Rosetta installed, scoring can be run for a single structure* baseline = **rs.io.get_sequence_and_structure** (‘4yod.pdb’)
	experiments = [‘no_target’, ‘static’, ‘pack’, ‘packmin’] scores = [‘score’, ‘LocalRMSDH’, ‘post_ddg’, ‘bb_clash’] scorename = [‘score’, ‘RMSD’, ‘ddG’, ‘bb_clash’] for experiment in experiments: * # Load Rosetta silent file from decoy generation* ds = **rs.io.parse_rosetta_file**(experiment + ‘.design’) *# Load decoy evaluation from a pre-processed CSV file.* *# Casting pd. DataFrame into DesignFrame is as easy as shown here.* ev = **rs.components. DesignFrame**(pd.read_csv(experiment + ‘.evals’)) *# Different outputs for the same decoys can be combined through* *# their ‘description’ field (decoy identifier)* df.append(ds.merge (ev, on = ‘description’)) *# Tables can be joined together into a single working object* df = pd.concat(df) # As we are comparing over BINDI’s sequence, that is our reference. df**.add_reference_sequence**(‘B’, baseline.iloc[0]**.get_sequence**(‘B’)[:-1])
Plot	fig = plt.figure (figsize = (170 / 25.4, 170 / 25.4)) grid = (12, 4) *# Show the distribution for key score terms* axs = **rs.plot.multiple_distributions** (df, fig, grid, values = scores, rowspan = 3, labels = scorename, x = ‘binder_state’, order = experiments, showfliers = False)
	*# Sequence score for a selected decoys with standard-matrix weights* ax = plt.subplot2grid(grid, (3, 0), fig = fig, colspan = 4, rowspan = 4) qr = df[df[‘binder_state’] == ‘no_target’].sort_values(‘score’).iloc[0] **rs.plot.per_residue_matrix_score_plot** ( *qr* , ‘B’, ax, ‘BLOSUM62’, add_alignment = False, color = 0) qr = df[df[‘binder_state’] == ‘no_pack’].sort_values(‘score’).iloc[0] **rs.plot.per_residue_matrix_score_plot** (qr, ‘B’, ax, ‘BLOSUM62’, add_alignment = False, color = 2, selections = [(‘43–64’, ‘red’)]) *# Small functions help edit the plot display* **rs.utils.add_top_title** (ax, ‘no_target (blue) - pack (green)’)
	*# Evaluate the variability of residue types in the binding region* ax = plt.subplot2grid(grid, (7, 0), fig = fig, colspan = 2, rowspan = 4) qr = df[df[‘binder_state’] == ‘no_target’] **rs.plot.sequence_frequency_plot** (qr, ‘B’, ax, key_residues = ‘43–64’, cbar = False, clean_unused = 0.1, xrotation = 90) **rs.utils.add_top_title** (ax, ‘no_target’) ax = plt.subplot2grid(grid, (7, 2), fig = fig, colspan = 2, rowspan = 4) ax_cbar = plt.subplot2grid(grid, (11, 0), fig = fig, colspan = 4) **rs.plot.sequence_frequency_plot** (df[df[‘binder_state’] == ‘pack’], ‘B’, ax, key_residues = ‘43–64’, cbar_ax = ax_cbar, clean_unused = 0.1, xrotation = 90) **rs.utils.add_top_title** (ax, ‘pack’)
	plt.tight_layout() plt.savefig(‘BMC_Fig5.png’, dpi = 300)

The code shows how to join data from multiple Rosetta experiments to assess the key difference between four design populations in terms of different scoring metrics and sequence recovery. Code comments are presented in italics while functions from the rstoolbox are highlighted in bold. Styling commands are skipped to facilitate reading, but can be found in the repository’s notebook.

## Discussion

The analysis of protein structures is an important approach to enable the understanding of fundamental biological processes, as well as, to guide design endeavours where one can alter and improve the activity and stability of newly engineered proteins for a number of important applications. In the age of massive datasets, structural data is also quickly growing both through innovative experimental approaches and more powerful computational tools. To deal with fast-growing amounts of structural data, new analysis tools accessible to users with beginner-level coding experience are urgently needed. Such tools are also enabling for applications in CPD, where large amounts of structural and sequence data are routinely generated. Here, we describe and exemplify the usage of rstoolbox to analyse CPD data illustrating how these tools can be used to distil large structural datasets and produce intuitive graphical representations.

CPD approaches are becoming more popular and achieving important milestones in generating proteins with novel functions [1]. However, CPD pipelines remain technically challenging with multiple design and selection stages which are different for every design problem and thus often require user intervention. Within the applications of rstoolbox, several functionalities can aid in this process, by providing an easy programmatic interface to perform selections, comparisons with native proteins, graphical representations and informing follow-up rounds of design in iterative, multi-step protocols. The tools presented here were devised for Rosetta CPD calculations, nevertheless the table-like data structure used allows for the easy creation of parsers for other protein modelling and design tools. This is especially relevant in other modelling protocols that require large sampling such as protein docking [33]. Importantly, rstoolbox can also be useful for structural bioinformatics and the analysis of structural features which have become more enlightening with the growth of different structural databases (e.g. PDB [34], SCOP [35], CATH [31]).

## Conclusion

Here, we present the rstoolbox, a Python library for the analysis of large-scale structural data tailored for CPD applications and adapted to a wide variety of user expertise. We endowed rstoolbox with an extensive documentation and a continuous integration setup to ensure code stability. Thus, rstoolbox can be accessed and expanded by users with beginner’s level programming experience guaranteeing backward compatibility. The inclusion of rstoolbox in design, protocol development and structural bioinformatics pipelines will aid in the comprehension of the human-guided decisions and actions taken during the processing of large structural datasets, helping to ensure their reproducibility.

## Availability and requirements

Project name: rstoolbox.

Project home page: https://lpdi-epfl.github.io/rstoolbox

Operating system(s): Tested on Linux and macOS.

Programming language: Python.

Other requirements: python2.7 or python3.4+. Non-standard Python libraries required are automatically installed during setup with pip.

License: MIT.

Any restrictions to use by non-academics: None.

## Abbreviations

CPD: Computational protein design
FunFolDes: Rosetta functional folding and design
RMSD: Root Mean square deviation
